# Wenxin Keli versus Sotalol for Paroxysmal Atrial Fibrillation Caused by Hyperthyroidism: A Prospective, Open Label, and Randomized Study

**DOI:** 10.1155/2015/101904

**Published:** 2015-05-17

**Authors:** Zhaowei Meng, Jian Tan, Qing He, Mei Zhu, Xue Li, Jianping Zhang, Qiang Jia, Shen Wang, Guizhi Zhang, Wei Zheng

**Affiliations:** ^1^Department of Nuclear Medicine, Tianjin Medical University General Hospital, Anshan Road No. 154, Heping District, Tianjin 300052, China; ^2^Department of Endocrinology, Tianjin Medical University General Hospital, Tianjin 300052, China

## Abstract

We aimed to compare effectiveness of Wenxin Keli (WK) and sotalol in assisting sinus rhythm (SR) restoration from paroxysmal atrial fibrillation (PAF) caused by hyperthyroidism, as well as in maintaining SR. We randomly prescribed WK (18 g tid) or sotalol (80 mg bid) to 91 or 89 patients. Since it was not ethical not to give patients antiarrhythmia drugs, no control group was set. Antithyroid drugs were given to 90 patients (45 in WK group, 45 in sotalol group); ^131^I was given to 90 patients (46 in WK group, 44 in sotalol group). Three months later, SR was obtained in 83/91 or 80/89 cases from WK or sotalol groups (*P* = 0.762). By another analysis, SR was obtained in 86/90 or 77/90 cases from ^131^I or ATD groups (*P* = 0.022). Then, we randomly assigned the successfully SR-reverted patients into three groups: WK, sotalol, and control (no antiarrhythmia drug was given) groups. After twelve-month follow-up, PAF recurrence happened in 1/54, 2/54, and 9/55 cases, respectively. Log-Rank test showed significant higher PAF recurrent rate in control patients than either treatment (*P* = 0.06). We demonstrated the same efficacies of WK and sotalol to assist SR reversion from hyperthyroidism-caused PAF. We also showed that either drug could maintain SR in such patients.

## 1. Introduction

Atrial fibrillation is the most common cardiac rhythm disturbance, increasing in prevalence with age. By definition, atrial fibrillation is a supraventricular tachyarrhythmia characterized by uncoordinated atrial activation with consequent deterioration of atrial mechanical function [1–3]. Clinicians should distinguish a first-detected episode of atrial fibrillation, whether or not it is symptomatic or self-limited. Patients with atrial fibrillation have markedly reduced survival rate compared with subjects without atrial fibrillation. In paroxysmal atrial fibrillation (PAF), sudden repeated changes in rhythm cause symptoms which most patients find very debilitating. In addition, PAF carries an increasing risk of thromboembolic events, when compared with chronic atrial fibrillation [4, 5]. Therefore, the effective treatment and prevention of this kind of arrhythmia has important clinical significance [1–3, 6, 7]. Atrial fibrillation occurs in 10% to 25% of patients with hyperthyroidism, more commonly in men and elderly patients [2, 8, 9]. Mainstay treatment is restoration of euthyroid state, which can be accomplished by antithyroid drugs, ^131^I, and surgery. Successful management of hyperthyroidism could result in restoration of sinus rhythm (SR) in up to two-thirds of patients [10]. Mechanism of hyperthyroidism-induced atrial fibrillation has been proposed [10–12]. It is generally agreed that shortening of action potential duration and effective refractory period play key roles in this electrophysiological abnormality.

Wenxin Keli (WK) is a pure Chinese herb medicine. It has been reported to be useful in the treatment of atrial fibrillation [13–15], ventricular arrhythmia [16, 17], myocardial infarction-induced arrhythmia, heart failure, Brugada syndrome [18], and so forth. WK extract is composed of 5 components:* Nardostachys chinensis* Batal. extract (NcBe),* Codonopsis*, notoginseng, amber, and rhizoma polygonati. Burashnikov and colleagues [13] recently presented a fascinating electrophysiological investigation of WK on atrial fibrillation. This study showed that WK, as a novel atrial-selective sodium-channel blocking agent, could prolong action potential duration and effective refractory period. This investigation was hailed in the same issue's editorial commentary as an emblematic milestone of integrating traditional Chinese medicine into Western medical practices [14]. In fact, WK monotherapy or in a combined antiarrhythmic regimen has been widely used for arrhythmia management in China. Chen and colleagues [15] recently conducted a meta-analysis and found solid evidence to prove WK as an effective drug to improve P-wave dispersion as well as to maintain SR in patients with PAF and its complications. However, the effect of WK on hyperthyroidism-induced atrial fibrillation has never been studied so far.

Therefore, in this open label and randomized study, we aimed to prospectively compare the effectiveness between WK and sotalol in assisting SR reversion from hyperthyroidism-caused PAF. We also intended to study their effectiveness in the maintenance of SR. Sotalol was chosen as a comparing drug, because it was proven to have efficacy to restore and maintain SR from atrial fibrillation. And sotalol possessed both class II and class III antiarrhythmic effects [2, 3].

## 2. Patients and Methods

### 2.1. Patients

From January 2011 till January 2013, a series of 180 hyperthyroidism patients (diagnosed as Graves' disease), who came to either Nuclear Medicine Department or Endocrinology Department, were consecutively enrolled in this prospective study. All of the patients had symptomatic PAF. There were 98 males (55.48 ± 12.02 years old) and 82 females (56.12 ± 9.98 years old). Entry criteria included PAF due to hyperthyroidism; electrocardiographic evidence of atrial fibrillation; symptoms such as palpitations, light headedness, chest pain, and dyspnoea in association with PAF; good compliance. Exclusion criteria were PAF due to other reasons, recent myocardial infarction, heart failure, inflammation such as pneumonia and diarrhea, unstable hepatic or renal function, poor compliance, and other major medical problems that would leave the patient with a life expectancy of less than two years. All enrolled patients gave their informed consent. This study was approved by the Institutional Review Board of Tianjin Medical University General Hospital (approval number #20101207A).

### 2.2. Definition

The diagnosis of PAF was made according to the American College of Cardiology Foundation/American Heart Association Task Force guideline definition; briefly, PAF had episodes that were generally less than 7 days (most less than 24 h), yet it was usually recurrent [1, 2].

### 2.3. Protocol

This study was designed as a prospective, open label, and randomized investigation. Generally, patients eligible for the study were allocated to one of the treatments using a computer generated random number algorithm. As reported [15], the clinical applications of WK against PAF include two aspects: restoration of SR from PAF and maintenance of SR afterwards. Therefore, we divided our study into two stages of sinus restoration and maintenance, in order to determine WK's effects on these two aspects.

Initially, baseline demographic data were obtained from the subjects. Relevant symptoms, cardiac diagnoses, and medical history were noted. Physical examination, 24-hour ambulatory electrocardiograph and/or regular 12-lead electrocardiograph, and serum biochemical tests (including electrolytes and renal and liver function) were carried out. All electrocardiographic recordings were reviewed by at least two experienced observers.

In the first part of the study, we randomly prescribed WK (18 g tid) or sotalol (80 mg bid) to 91 patients (49 males, 42 females) or 89 patients (49 males, 40 females), respectively. This part of the study compared the effectiveness of WK and sotalol to restore SR from PAF. In this investigation, it was not ethical not to give the patients any antiarrhythmia drugs. So, we did not design control; we just compared WK and sotalol. Antithyroid drugs (ATD) were given to 90 patients (45 in WK group, 45 in sotalol group), and ^131^I was also given to 90 patients (46 in WK group, 44 in sotalol group). Due to the similar ethical reason, no control group was set. ATD-treated patients were given methimazole (initial dose 30 mg per day). ^131^I therapeutic procedure was performed according to our protocol [19, 20]. Thyroid radioiodine uptake value was measured at 6, 24, 48, and 72 hours after an oral tracer dose uptake of ^131^I (about 74 kBq) by a nuclear multifunctional instrument (MN-6300XT Apparatus, Technological University, China). Then ^131^I effective half-life time (*T*_1/2eff_) and maximum uptake in thyroid were calculated. Thyroid ultrasonography was performed by using a color doppler ultrasound machine (GE Vingmed Ultrasound Vivid Five, Horten, Norway). Thyroid volume was calculated with the following formula: volume (cm^3^) = (width × length × thickness of left lobe) + (width × length × thickness of right lobe). Thyroid weight (g) = 0.479 × volume (cm^3^). Serum thyroid hormones were tested by an immunofluorometric assay, including free triiodothyronine (FT3, reference 3.50–6.50 pmol/L), free thyroxine (FT4, reference 11.50–23.50 pmol/L), and thyroid stimulating hormone (TSH, reference 0.20–5.00 *μ*IU/mL). The therapeutic dose of ^131^I was calculated as the following formula [19, 20]: dose (37 MBq) = (thyroid weight (g) × absorption dose (Gy/g) × 0.67)/(*T*_1/2eff_ (days) × maximum uptake (%)). Absorption dose = 100 Gy/g thyroid tissue; 0.67 is a rectified factor. Participants visited our outpatient department every month. At each scheduled follow-up visit, physical examination and routine laboratory tests were done. And, at the end of the third month, ambulatory electrocardiograph and/or regular 12-lead electrocardiograph were repeated; all relevant symptoms were documented. Disappearing of PAF was defined as restoration of SR.

In the second part of the study, we randomly assigned the successfully SR-reverted patients into one of the following three groups: 54 cases were given WK (9 g tid), 54 cases were given sotalol (40 mg bid), and 55 cases served as control. In this part of the study, the control patients did not take any antiarrhythmia drug. Since patients recruited at this stage had much better improved thyroid status, and all of them were in SR when entering this investigation, it was ethically approved by our Institutional Review Board not to give the control patients any antiarrhythmia drugs. If patients were still in hyperthyroidism status, appropriate dose of methimazole was given to maintain euthyroidism. If the patients were in posttherapeutic hypothyroidism status, appropriate dose of levothyroxine was given to maintain euthyroidism. For hypothyroid patients who had already restored SR, WK and sotalol were stopped. Participants were asked to visit our outpatient department every three months. At each scheduled or sometimes unscheduled follow-up visit, physical examination and routine laboratory tests were repeated. And, at the end of the twelfth month, ambulatory electrocardiograph and/or regular 12-lead electrocardiograph were done; all relevant symptoms were documented. Time-point of PAF recurrence, its frequency, and related symptoms were collected as well.

**Figure 1:**
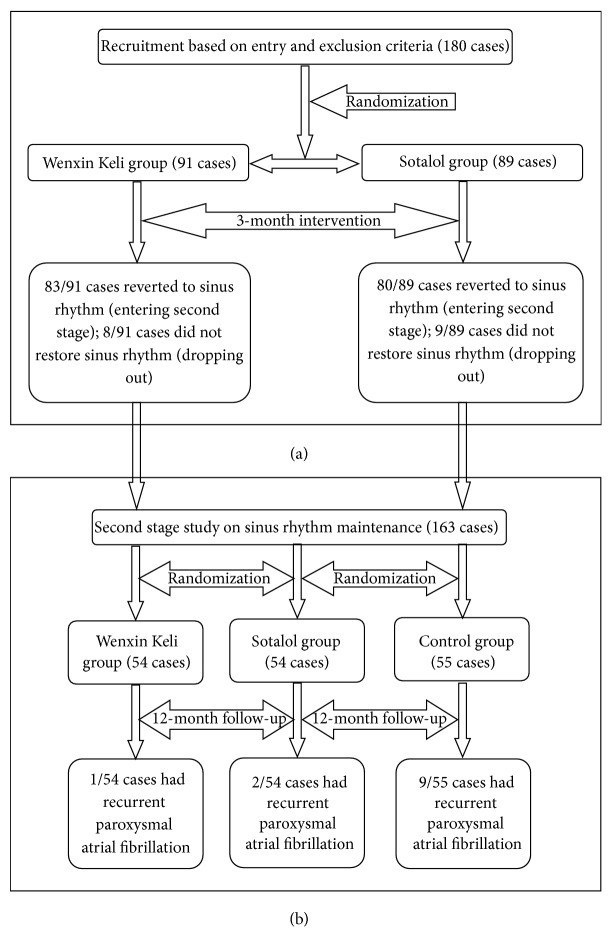
Participant flow chart. Initially, in the first stage of the study (a), 180 eligible hyperthyroidism patients with paroxysmal atrial fibrillation were randomized into either Wenxin Keli (91 cases) or sotalol (89 cases) treatment for sinus rhythm restoration. At the end of the first stage intervention, 83/91 cases and 80/89 cases were reverted to sinus rhythm, respectively. There were 8/91 cases and 9/89 cases who did not restore sinus rhythm. These 17 patients (still with atrial fibrillation) were not eligible for the second part of the study, and they were dropped out. In the second stage of the study (b), all sinus rhythm reverted patients (163 cases) were randomized into one of the following three groups: WK (54 cases), sotalol (54 cases), and control (55 cases) groups. The purpose is to observe drug's sinus rhythm maintenance effect. At the end of the second stage intervention, 1/54 cases, 2/54 cases, and 9/55 cases had recurrent paroxysmal atrial fibrillation, respectively.

Participant flow chart was presented in Figure 1 to illustrate the whole study process for better understanding.

### 2.4. Statistical Analysis

All data were presented as mean ± SD. Statistics were performed with SPSS 17.0 (SPSS Incorporated, IL, USA). Differences between two groups were analyzed by independent samples *t*-test. Differences between multiple groups were analyzed by one-way analysis of variance (ANOVA), and then least significant difference (LSD) test was used for multiple comparisons among the groups.  *χ* ^*2*^ test was adopted to determine case number changes of patients after different treatments.  *χ* ^*2*^ test was also used to check whether sex had a significant influence on the intergroup differences. Kaplan-Meier analysis by Log-Rank *χ* ^*2*^ test was used to estimate the cumulative recurrent rate of PAF in different groups.* P* value not exceeding 0.05 was considered statistically significant.

## 3. Results

### 3.1. Sinus Rhythm Restoration by Different Therapies

**Figure 2:**
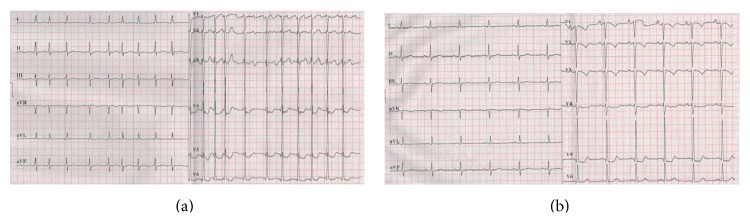
A typical case of successful sinus rhythm restoration from paroxysmal atrial fibrillation. A 64-year-old male patient was diagnosed with Graves' disease for eight years. He had paroxysmal atrial fibrillation for three years (a). He was given 6 mCi of ^131^I for the treatment of Graves' disease. And Wenxin Keli (18 g tid) was prescribed during and after the ^131^I treatment. Baseline free triiodothyronine, free thyroxine, and thyroid stimulating hormone were 21.46 pmol/L, 104.8 pmol/L, and 0.011 *μ*IU/mL, respectively. One month later, when sinus rhythm was restored (b), free triiodothyronine, free thyroxine, and thyroid stimulating hormone were 3.35 pmol/L, 12.89 pmol/L, and 4.52 *μ*IU/mL, respectively. At the three-month end-point of the first investigation, thyroid hormones were still normal. After entering the second investigation, Wenxin Keli (9 g tid) was prescribed during the follow-up. His thyroid function maintained normal level, and his heart rhythm maintained sinus rhythm during the rest of the study.

First, baseline information revealed no significant differences of hyperthyroidism history, PAF history, or thyroid hormone levels between the groups (Table 1). Data in this investigation were analyzed by two ways. In the first analysis, three months after treatment of WK or sotalol, SR was obtained in 83/91 cases (91.209%) or 80/89 cases (89.888%); *χ* ^*2*^ test showed no significant differences, indicating equal efficacies of the two drugs for assisting SR reversion (Table 2). Sex did not cause significant differences between the groups (Table 2). Thyroid hormones also demonstrated no differences before or after treatments (Table 3). In the second analysis, after treatment of ^131^I or ATD, SR was obtained in 86/90 cases or 77/90 cases; *χ* ^*2*^ test showed significant differences, indicating better effects of ^131^I treatment (Table 4). Thyroid hormones displayed no differences before treatment, yet significant differences existed after treatment (Table 5). A typical case showing successful converted SR from PAF was presented (Figure 2).

### 3.2. Sinus Rhythm Maintenance by Different Therapies

**Figure 3:**
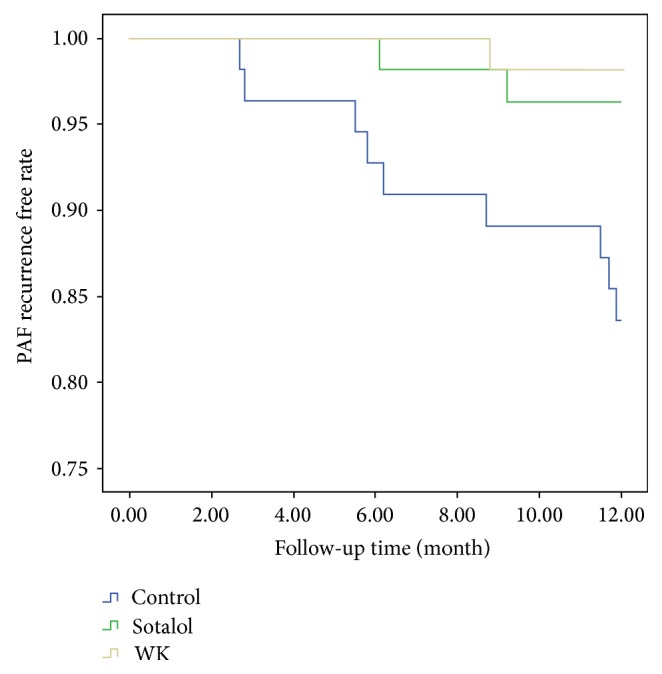
The cumulative recurrent rate of paroxysmal atrial fibrillation during the follow-up in different groups. In the second part of the study, we randomly assigned the successfully sinus rhythm reverted patients into one of the following three groups: 54 cases were given Wenxin Keli (9 g tid), 54 cases were given sotalol (40 mg bid), and 55 cases served as control. Kaplan-Meier analysis by Log-Rank *χ* ^*2*^ test was used to determine the cumulative recurrent rate of paroxysmal atrial fibrillation in different groups during the one-year-long follow-up. Vertical axle was PAF recurrence free rate and horizontal axle was the follow-up time (WK = Wenxin Keli, PAF = paroxysmal atrial fibrillation).

Data in the second investigation were analyzed by two methods. First, at the end of twelve-month follow-up, recurrent PAF happened in 1/54 (1.852%), 2/54 (3.704%), and 9/55 (16.364%) cases in WK, sotalol, or control groups, respectively. We found no differences of thyroid hormones at any follow-up time-point among the groups (Table 6). However, *χ* ^*2*^ test showed significant differences between WK and control groups and significant differences between sotalol and control groups, while there were no differences between WK and sotalol groups (Table 7). Second, Kaplan-Meier curves were drawn to determine the cumulative recurrent rate of PAF in different groups (Figure 3). Log-Rank test showed significant higher PAF recurrent rate in control patients compared with either treatment (*χ* ^2^ = 10.229, *P* = 0.06). Therefore, we proved that both WK and sotalol could successfully maintain SR.

### 3.3. Side Effects

Since there is always an inherent bitter taste in Chinese medicine, some patients would unavoidably complain about the gastrointestinal discomfort or related symptoms after taking WK. Altogether, there were 10/91 cases (10.989%) in the first investigation and 6/54 (11.111%) in the second investigation who reported various degrees of nausea and dizziness after taking WK. However, all patients showed endurance and continued with the medication. For sotalol groups, the gastrointestinal discomfort was far less frequent; there were only 3/89 cases (3.371%) in the first investigation and 2/54 (3.704%) in the second investigation who reported mild stomach discomfort. However, after taking sotalol, 2/89 cases (2.247%) in the first investigation developed symptomatic bradycardia, whose PAF disappeared though. The problems completely dissolved after dose reduction from 80 mg bid to 40 mg bid for one patient and from 80 mg bid to 40 mg qd for the other patient. These two patients' heart rhythm maintained SR during the rest of the study. WK showed no bradycardia side effect. No other unwanted incidences were recorded.

## 4. Discussion

The risk of developing atrial fibrillation in patients with hyperthyroidism is approximately 6-fold of the euthyroidism population, which aggravates the overall conditions of such patients [9]. Successful treatment of hyperthyroidism with either ^131^I or ATD is associated with a reversion to SR in a majority of patients [10, 21, 22]. However, pharmacological management of atrial fibrillation in patients with hyperthyroidism is still an issue lacking in comprehensive analysis. In general, rate control is very important to reduce the mortality rate of patients with atrial fibrillation [6, 7]. Selective or nonselective *β*-blockers can provide rapid symptom relief by reducing the ventricular rate, but these agents are unlikely to convert PAF to SR. Pharmacotherapy of atrial fibrillation has an advantage over electrical cardioversion and the catheter ablation methods, because it can be used on an outpatient basis [23]. However, the optimal pharmacological means to restore and maintain SR in patients with hyperthyroidism-caused atrial fibrillation remains controversial.

WK is identified as a novel drug against atrial fibrillation. Its mechanism has been elucidated recently. Burashnikov and colleagues [13] have implemented an isolated canine perfused right atrial preparation and recorded atrial and ventricular transmembrane action potentials and pseudoelectrograms before and after intracoronary perfusion of various concentrations of WK. Interestingly, WK produced effects more noticeable in atrial tissue than in ventricular tissue, as it caused action potential duration shortening and prolongation of effective refractory periods in an atrial-selective manner. In addition, WK produced a greater reduction in the maximum rate of rise of the action potential upstroke and a larger increase in the diastolic threshold for excitation in atrial cells, suggestive of sodium-channel current blockade. This was confirmed in HEK293 cells expressing the sodium ion channel protein SCN5A, in which WK decreased the peak sodium-channel current, in both dose-dependent and use-dependent fashions. Finally, antiarrhythmic properties of WK were illustrated by the prolongation of the P-wave duration and both the prevention and termination of acetylcholine-mediated atrial fibrillation. The above mechanism of WK acts directly against the electrophysiological changes in hyperthyroidism-induced atrial fibrillation [10–12].

Contrary to the relatively new discovery of the MK mechanisms, traditional Chinese medicines were first documented about 2500 years ago by Confucian scholars and are now still being used by tens of millions in China as well as around the world [14, 24, 25]. Clinical evidence of WK is based on results of clinical trials being carried out in Chinese hospitals for years. These studies have shown that WK can significantly improve heart palpitations, chest tightness, shortness of breath, fatigue, insomnia, and other symptoms of atrial fibrillation [15]. Currently, WK monotherapy or combined therapy with antiarrhythmic drugs has been recommended as an effective method for atrial fibrillation in China. In fact, WK is the first Chinese-developed antiarrhythmic medicine to be approved by the Chinese state. Besides antiarrhythmic property, clinical trials have also confirmed that WK can increase coronary blood low, reduce myocardial oxygen consumption, enhance myocardial compliance, improve myocardial hypoxia tolerance, relieve anterior and posterior cardiac loading, and reduce myocardial tissue damage in patients with high blood pressure. These clinical evidences are in accordance with WK's basic mechanistic research findings recently [13, 16–18].

In the current investigation, we provided the first clinical evidence of WK as well as sotalol on the management of hyperthyroidism-induced PAF in two aspects. First, the drugs could assist SR reversion from PAF caused by hyperthyroidism. Second, the drugs could maintain SR afterwards. The second application seemed more important, since the first application was very dependent on the degree of thyroid hormone reduction. We showed that there were nearly the same efficacies of both WK and sotalol to assist SR restoration. However, ^131^I was much more effective for hyperthyroidism management and thereafter to gain better SR reversion. We believed that this was largely due to better therapeutic results of ^131^I to control thyroid hormones (Tables 4 and 5). In the latter investigation, we showed that both WK and sotalol could maintain SR with equal abilities in our cohort, who have already gained SR after treatments. The cumulative recurrent rate was significantly lower in the drug-treated patients than in the control cases (Figure 3). Our study proved the usefulness and effectiveness of WK as well as sotalol on the long-term maintenance management of such patients, which is indeed very important for clinical purposes.

Although WK's effect on hyperthyroidism-related atrial fibrillation has never been reported before, WK's anti-atrial-fibrillation ability is not new discovery. All of WK's clinical studies are published in Chinese language so far; however, after considering their relevancy to the current study, further comments are deserved. Chen and colleagues [15] compiled and evaluated all available randomized controlled trials regarding WK's therapeutic effects against PAF (complicated with diseases other than hyperthyroidism) according to the PRISMA systematic review standard. There were nine trials analyzing therapeutic effectiveness of WK alone or combined with Western medicine, compared with no medicine or Western medicine alone, in patients with PAF [26–34]. Most of the trials used amiodarone as the Western medicine, which cannot be used for hyperthyroidism-related atrial fibrillation. These trials were not homogeneous, requiring the use of the random effects model for statistical analysis. Meta-analysis results demonstrated a significant difference between the two therapeutic groups (the WK combination therapy was much better). Seven trials used the maintenance rate of SR at six months following treatment as an outcome measurement [33–39]. These seven trials compared the combination of WK plus Western medicine with Western medicine alone (mostly amiodarone). These trials were homogeneous, requiring the use of the fixed effects model for statistical analysis. The rate of maintenance of SR in the former group was greater than the latter group. Meta-analysis results showed that there was a significant beneficial effect in the WK combination regimens compared with the Western medicine monotherapy. The above literature is in conformity with our findings in that WK is an effective drug for the management of PAF, not only for initial SR reversion therapy but also for the long-term maintenance therapy.

In conclusion, we demonstrated the same efficacies of WK and sotalol to assist SR reversion from hyperthyroidism-related PAF. ^131^I was better to control thyroid hormone and to gain SR reversion. We also showed that both WK and sotalol could maintain SR with equal abilities in those PAF hyperthyroidism patients who had already gained SR after treatments. Therefore, WK is a useful drug that should be advocated in the initial treatment of PAF caused by hyperthyroidism, as well as in the follow-up management strategy.

## Figures and Tables

**Table 1:** Baseline information of all participants.

Parameters	WK^*^ treatment (91 cases)	Sotalol treatment (89 cases)	*t* value (*P* value)^**^
Hyperthyroidism history (years)	8.374 ± 2.619	8.551 ± 2.680	0.448 (0.655)
PAF^*^ history (years)	4.099 ± 1.599	4.213 ± 1.675	0.469 (0.639)
FT3^*^ (pmol/L)	24.613 ± 5.059	24.405 ± 5.006	−0.278 (0.781)
FT4^*^ (pmol/L)	118.697 ± 29.213	116.132 ± 28.266	−0.598 (0.550)
TSH^*^ (*μ*IU/mL)	0.007 ± 0.010	0.009 ± 0.015	1.191 (0.235)

^∗^WK: Wenxin Keli, PAF: paroxysmal atrial fibrillation, FT3: free triiodothyronine, FT4: free thyroxine, and TSH: thyroid stimulating hormone; ^**^analyzed by independent samples *t*-test.

**Table 2:** Case number distribution of patients after WK^*^ or sotalol treatments in the first investigation.

Groups (case number)	Male		Female	
	Total number	SR^*^ restored number	Total number	SR^*^ restored number
WK^*^ treatment (91 cases)	49	45	42	38
Sotalol treatment (89 cases)	49	44	40	36

*χ* ^2^ value (*P* value)_(WK) : (sotalol)_^∗∗^	0.092 (0.762)			
*χ* ^2^ value (*P* value)_(male) : (female)_^∗∗^	0.017 (0.896)			

^∗^WK: Wenxin Keli; SR: sinus rhythm; ^**^analyzed by *χ* ^2^ test.

**Table 3:** Comparisons of thyroid hormones in patients before and after WK^*^ or sotalol treatments in the first investigation.

Before treatments			
	WK^*^ treatment (91 cases)	Sotalol treatment (89 cases)	*t* value (*P* value)^**^
FT3^*^ (pmol/L)	24.613 ± 5.059	24.405 ± 5.006	−0.278 (0.781)
FT4^*^ (pmol/L)	118.697 ± 29.213	116.132 ± 28.266	−0.598 (0.550)
TSH^*^ (*μ*IU/mL)	0.007 ± 0.010	0.009 ± 0.147	1.191 (0.235)

Three months after treatments			
	WK^*^ treatment (91 cases)	Sotalol treatment (89 cases)	*t* value (*P* value)^**^

FT3^*^ (pmol/L)	6.495 ± 3.713	6.596 ± 3.740	0.182 (0.855)
FT4^*^ (pmol/L)	21.447 ± 11.727	21.655 ± 10.612	0.125 (0.901)
TSH^*^ (*μ*IU/mL)	6.210 ± 10.002	5.752 ± 8.915	−0.324 (0.746)

^∗^WK: Wenxin Keli, FT3: free triiodothyronine, FT4: free thyroxine, and TSH: thyroid stimulating hormone; ^**^analyzed by independent samples *t*-test.

**Table 4:** Case number distribution of patients after ^131^I or ATD^*^ treatments in the first investigation.

Groups (case number)	Male		Female	
	Total number	SR^*^ restored number	Total number	SR^*^ restored number
^131^I treatment (90 cases)	49	47	41	39
ATD^*^ treatment (90 cases)	49	42	41	35

*χ* ^2^ value (*P* value)_(^131^I) : (ATD)_^∗∗^	5.262 (0.022)			
*χ* ^2^ value (*P* value)_(male) : (female)_^∗∗^	0.017 (0.896)			

^∗^ATD: antithyroid drugs; SR: sinus rhythm; ^**^analyzed by *χ* ^2^ test.

**Table 5:** Comparisons of thyroid hormones in patients before and after ^131^I or ATD^*^ treatments in the first investigation.

Before treatments			
	^131^I treatment (90 cases)	ATD^*^ treatment (90 cases)	*t* value (*P* value)^**^
FT3^*^ (pmol/L)	24.056 ± 5.321	24.964 ± 4.685	1.215 (0.226)
FT4^*^ (pmol/L)	117.633 ± 29.225	117.225 ± 28.322	−0.095 (0.924)
TSH^*^ (*μ*IU/mL)	0.007 ± 0.011	0.009 ± 0.014	0.757 (0.450)

Three months after treatments			
	^131^I treatment (90 cases)	ATD^*^ treatment (90 cases)	*t* value (*P* value)^**^

FT3^*^ (pmol/L)	5.837 ± 2.830	7.252 ± 4.330	2.595 (0.010)
FT4^*^ (pmol/L)	19.378 ± 8.292	23.722 ± 13.120	2.655 (0.009)
TSH^*^ (*μ*IU/mL)	6.427 ± 9.702	5.539 ± 9.237	−0.629 (0.530)

^∗^ATD: antithyroid drugs, FT3: free triiodothyronine, FT4: free thyroxine, and TSH: thyroid stimulating hormone; ^**^analyzed by independent samples *t*-test.

**Table 6:** Comparisons of thyroid hormones at any follow-up time-points in the second investigation.

Baseline				
	WK^*^ treatment (54 cases)	Sotalol treatment (54 cases)	Control (55 cases)	*F* value (*P* value)^**^
FT3^*^ (pmol/L)	5.532 ± 2.372	5.752 ± 2.608	5.680 ± 2.486	0.110 (0.896)
FT4^*^ (pmol/L)	18.469 ± 7.182	19.351 ± 7.577	19.046 ± 7.576	0.195 (0.823)
TSH^*^ (*μ*IU/mL)	7.126 ± 10.449	5.859 ± 8.668	6.832 ± 10.110	0.249 (0.780)

Three months				
	WK^*^ treatment (54 cases)	Sotalol treatment (54 cases)	Control (55 cases)	*F* value (*P* value)^**^

FT3^*^ (pmol/L)	5.035 ± 0.934	5.129 ± 0.908	5.098 ± 0.965	0.140 (0.870)
FT4^*^ (pmol/L)	15.664 ± 3.112	16.061 ± 3.336	15.994 ± 3.465	0.222 (0.801)
TSH^*^ (*μ*IU/mL)	4.683 ± 4.211	4.083 ± 3.456	4.352 ± 3.885	0.326 (0.722)

Six months				
	WK^*^ treatment (54 cases)	Sotalol treatment (54 cases)	Control (55 cases)	*F* value (*P* value)^**^

FT3^*^ (pmol/L)	5.257 ± 0.930	5.373 ± 0.915	5.381 ± 1.057	0.277 (0.758)
FT4^*^ (pmol/L)	16.446 ± 3.339	16.916 ± 3.727	16.955 ± 4.014	0.317 (0.729)
TSH^*^ (*μ*IU/mL)	4.032 ± 3.492	3.567 ± 2.885	3.756 ± 3.202	0.288 (0.750)

Nine months				
	WK^*^ treatment (54 cases)	Sotalol treatment (54 cases)	Control (55 cases)	*F* value (*P* value)^**^

FT3^*^ (pmol/L)	5.367 ± 0.975	5.458 ± 0.958	5.590 ± 1.328	0.566 (0.569)
FT4^*^ (pmol/L)	17.184 ± 3.208	17.760 ± 4.131	18.084 ± 4.833	0.668 (0.514)
TSH^*^ (*μ*IU/mL)	2.912 ± 1.730	2.701 ± 1.666	2.785 ± 1.719	0.211 (0.810)

Twelve months				
	WK^*^ treatment (54 cases)	Sotalol treatment (54 cases)	Control (55 cases)	*F* value (*P* value)^**^

FT3^*^ (pmol/L)	5.562 ± 0.969	5.740 ± 1.302	5.874 ± 1.406	0.866 (0.422)
FT4^*^ (pmol/L)	17.830 ± 3.485	18.532 ± 5.113	18.901 ± 5.388	0.716 (0.490)
TSH^*^ (*μ*IU/mL)	2.519 ± 1.420	2.388 ± 1.423	2.409 ± 1.488	0.129 (0.879)

^∗^WK: Wenxin Keli, FT3: free triiodothyronine, FT4: free thyroxine, and TSH: thyroid stimulating hormone; ^**^analyzed by one-way analysis of variance and least significant difference test.

**Table 7:** Cumulative recurrent PAF^*^ at the end of follow-up in the second investigation.

Groups (case number)	Male		Female	
	Total number	Cumulative recurrent PAF	Total number	Cumulative recurrent PAF
WK^*^ treatment (54 cases)	27	0	27	1
Sotalol treatment (54 cases)	27	2	27	0
Control (55 cases)	35	4	20	5

*χ* ^2^ value (*P* value)_(WK) : (control)_^∗∗^	6.886 (0.009)			
*χ* ^2^ value (*P* value)_(sotalol) : (control)_^∗∗^	4.813 (0.028)			
*χ* ^2^ value (*P* value)_(WK) : (sotalol)_^∗∗^	0.343 (0.558)			

^∗^WK: Wenxin Keli; PAF: paroxysmal atrial fibrillation; ^**^analyzed by *χ* ^2^ test.
